# Effects of electrochemical ageing of lithium-ion battery electrolyte on its in vitro genotoxicity: a special focus on sultones

**DOI:** 10.1007/s00204-025-04246-2

**Published:** 2026-01-14

**Authors:** Elisabeth Christine Muschiol, Louisa Sophie Tölke, Christian-Timo Lechtenfeld, Thorsten Kuczius, Martin Winter, Sascha Nowak, Melanie Esselen

**Affiliations:** 1 Institute of Food Chemistry, University of Münster, Corrensstraße 45, 48149 Münster, Germany; 2 MEET Battery Research Center, Institute of Physical Chemistry, University of Münster, Corrensstraße 46, 48149 Münster, Germany; 3 Institute of Hygiene, University Hospital Münster, Robert-Koch-Straße 41, 48149 Münster, Germany; 4 Forschungszentrum Jülich GmbH, Helmholtz-Institute Münster, IMD-4, Corrensstraße 46, 48149 Münster, Germany

**Keywords:** Lithium-ion batteries, Electrolyte, Electrochemical ageing, Mutagenicity, Genotoxicity

## Abstract

Lithium-ion batteries (LIBs) play a key role in the transformation of society away from fossil fuels. Despite extensive research on their behaviour and improvement, toxicological aspects are often neglected, in particular regarding the liquid organic electrolyte. This study aimed to investigate the genotoxic and mutagenic potential as well as cell cycle effects of LIB electrolytes including one of the three sulphur-containing additives 1,3‑propanesultone (PS), prop‑1‑ene‑1,3‑sultone (PES) and 1,3,2‑dioxathiolane‑2,2‑dioxide (DTD). These additives are used in LIB to improve performance. Genotoxicity was assessed in HepG2 cells by two different versions of the in vitro micronucleus assay, whereas for mutagenicity testing, the bacterial reverse mutation assay in *Salmonella typhimurium* strains was used. The study focussed mainly on the investigation of the electrolyte and its constituents in different electrochemical ageing stages. Pure additives and one suspected degradation product each were investigated as well. It was shown that the toxicity of the pristine electrolyte related to that of the present additive. However, ageing led to electrolyte decomposition and the formation of new species, which had a major influence on toxicity. In sum, the extent to which toxicity was reduced depended on the additive. The results suggested that for the application in LIBs, DTD should be preferred over PS and PES from a toxicological point of view.

**Supplementary Information:**

The online version contains supplementary material available at 10.1007/s00204-025-04246-2.

## Introduction

Many different approaches are being used in battery research to improve performance (Kasnatscheew et al. 2018; Wang et al. 2020; Zhang et al. 2022b; Chen et al. 2022). One strategy in state-of-the-art lithium-ion batteries (LIBs) with liquid organic electrolytes is introducing additives–small quantities of substances other than solvents and conducting salt–into the electrolyte (Abraham et al. 1984; Matsuda und Morita 1989; Tong et al. 2021). One of their many purposes is an enhanced stability of the solid electrolyte interphase (SEI) and/or cathode electrolyte interphase (CEI), which lead to improved performance, in particular higher safety, and longer cycle life (Peschel et al. 2023; Wang et al. 2020). Among additives that improve SEI and CEI formation, the sulphur-containing additives (Table 1) 1,3‑propanesultone (PS), prop‑1‑ene‑1,3‑sultone (PES) and 1,2‑ethylene sulphate (1,3,2-dioxathiolane‑2,2‑ dioxid, DTD) have played an important role in batteries since the early 2000s (Tong et al. 2021).

**Table 1:** Chemical structures and molar masses of the investigated electrolyte additives as well as classifications according to the International Agency for Research on Cancer (IARC 2017), the Scientific Committee on Occupational Exposure Limits (SCOEL 2013) and the regulation of the European Union for Classification, Labelling and Packaging (CLP, Regulation (EC) No 1272/2008). * probably carcinogenic to humans, ** presumed humand carcinogen, *** genotoxic carcinogen without threshold, n.l. not listed

	PS	PES	DTD
			
Molar mass	122.14 g/mol	120.13 g/mol	124.12 g/mol
IARC	category 2A *	n.l.	n.l.
SCOEL	category 1B **	n.l.	n.l.
CLP	category A ***	n.l.	n.l.

**Fig. 1:**
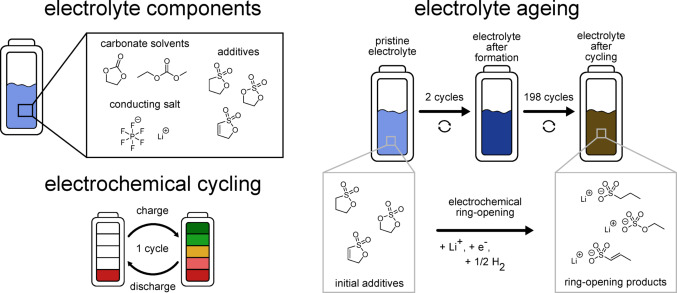
Illustration of the investigated electrolyte components, the principle of electrochemical cycling of a battery and an exemplary ageing process with the ageing stages and suspected degradation products

The drawback of the sulphur-containing additives is their toxicity, yet this is not always considered in battery research. PS is classified as carcinogenic by various scientific assessments and in legally binding classifications (Table 1), whereas PES and DTD are not listed due to missing data for comprehensive evaluation. However, there are studies which show carcinogenic effects in rodents for both PS (Druckrey et al. 1970; Ulland et al. 1971; van Duuren et al. 1971) and DTD (van Duuren et al. 1974). A case study of workers exposed to PS revealed a series of tumours comparable to those observed in animal studies, among them rare glioblastomas which coincide with a number of gliomas detected in animals (Bolt und Golka 2012, 2004). PS is capable of forming DNA-adducts (Goldschmidt et al. 1974; Hemminki 1983) which then lead to structural chromosomal aberrations, indicating clastogenic effects (Kaul 1985; Kim et al. 2020). Furthermore, PS and DTD are known mutagens (Braun et al. 1977); however, they are not stable in aqueous solution (Fischer 1964; Fischer et al. 1975). Hydroxypropanesulfonic acid (HPSA), the hydrolysed form of PS, is reported to be not mutagenic (Abe et al. 2014). This could lead to the conclusion that the use of PS is safe, as it comes in contact with humidity as soon as it is released from a battery. However, the present study aims to investigate the toxicity of the complex electrolyte mixture at different ageing stages of the battery (Fig. 1) rather than only considering the effect of the pure additives. Additionally, PES has hardly been researched toxicologically at all but is expected to produce similar effects as PS due to structural resemblance, as is DTD.

Toxicological concerns may arise not only from the additives used in the electrolyte, but also from the decomposition products formed during cycling. Peschel et al. (2023) have found that PS undergoes electrochemical ring-opening to form the ionic ageing product 1‑propanesulfonate (1-PS), which is shown in Fig. 1 and Fig. S1. In line with this proposed mechanism, possible ring-opening products of PES and DTD are additionally shown. These compounds can potentially be esterified during further cycling and subsequently show structural similarities to well-known genotoxins like methyl methane sulfonate (Peschel et al. 2023).

A common argument against the toxicological relevance of additives is their assumed low levels in LIBs. But even though the levels are low, there is a general need to replace CMR substances (carcinogenic, mutagenic, toxic for reproduction). Workers can come into contact with the electrolyte during manufacturing or recycling (Lu et al. 2023; Kang et al. 2023), which emphasizes the importance to investigate both the pristine and the electrochemically aged electrolyte. Previous studies have mostly focussed on the electrode materials rather than on the electrolyte itself (Kang et al. 2013; Qi et al. 2024; Wang et al. 2017; Sironval et al. 2020). Apart from occupational exposure, people are at risk of being exposed by accidents involving electric vehicles (EV). Even though there might be more severe hazards originating from a car crash, about 10 wt% of LIBs is the electrolyte that can be released and pose an additional thread to the passengers (Zhang et al. 2022a). Strehlau et al. (2017) incubated human lung adenocarcinoma cells with electrolyte and analysed the uptake of carbonates into the cells via gas chromatography–mass spectrometry and studied the transfer of the electrolyte components across the blood-cerebrospinal fluid barrier. However, to the best of our knowledge, there is no assessment of the toxicity of the spent electrolyte. Assuming that the additives are incorporated into passivation layers during cycling (Tong et al. 2021), the toxicity of the electrolyte should decrease. However, the impact of decomposition products is unclear. Therefore, this paper evaluated the genotoxic and mutagenic properties using the in vitro micronucleus (MN) assay and the Ames test, respectively, as well as cytotoxicity issues, of electrolyte with the three sulphur-containing additives PS, PES and DTD.

## Experimental section

### Chemicals and biological material

The chemicals and reagents were purchased from Sigma-Aldrich (Taufkirchen, Germany), Santa Cruz Biotechnology (Heidelberg, Germany), Carl Roth (Karlsruhe, Germany), Thermo Fisher Scientific (Schwerte, Germany) and TCI (Eschborn, Germany), if not specified otherwise. Purified water was created by reverse osmosis using a miniRo system (Veolia Water Technologies, Celle, Germany). The human hepatoblastoma cell line HepG2 (ACC 180) was obtained from DSMZ (German Collection of Microorganisms and Cell Cultures, Braunschweig, Germany). *Salmonella typhimurium* strains TA97a, TA98, TA100, TA1535 and TA1537 were obtained from hjs-consulting (Neuenburg am Rhein, Germany).

### Sample preparation

All electrolytes examined in this study were based on LP57 (ethylene carbonate (EC)/ethyl methyl carbonate (EMC) 3 + 7 (*v*/*v*) with 1 M lithium hexafluorophosphate (LiPF_6_), Solvionic, Toulouse, France). LP57 was analysed without additives (EL_LP57_) and with 2 wt% of PS, PES or DTD each (EL_PS_, EL_PES_ and EL_DTD_, respectively). Each electrolyte was investigated in its pristine form and after two and 200 charge–discharge cycles. Additionally, the additives and standards of associated ring-opening-products (Peschel et al. 2023) were studied as pure substances. In Table 2, the used electrolyte concentrations are related to the corresponding additive concentrations for the different assays. If possible, the studied amounts of the pure substances related to these concentrations.

**Table 2:** Electrolyte concentrations applied in the different experimental assays and the corresponding approximate additive concentrations (~ due to different molar masses). It has to be noted that these concentrations are strictly limited to the pristine electrolytes, as additive degradation occurs during cycling in different amounts. MN assay: micronucleus assay

	c (electrolyte) [ppm]		~	c (additive) [µM]
MN assay	250	~		40
	500	~		80
	2 500	~		400
	5 000	~		800
reverse mutation test	40	~		6.4
	200	~		32
	400	~		64
	2 000	~		320
	4 000	~		640
	20 000	~		3200

For electrochemical evaluations, commercially available multilayer-wound NMC622||Artificial Graphite (AG) pouch cells (Li‑FUN Technologies Ltd., China) with a nominal capacity of 200 mAh and optimized for operation up to 4.2 V were employed. Prior to assembly, the cells were dried at 80 °C under reduced pressure overnight. Each cell was subsequently filled with 745 µL of electrolyte (equivalent to 1.3 times the total pore volume) and sealed at 20% ambient pressure (GN‑HS350V, Gelong LIB Co., China) in a dry room with a dew point below − 42 °C. A wetting period of 10 h was applied before initiating electrochemical testing. During operation, a pressure of 2 bar was applied to the cell stacks using custom-designed cell holders.

The cell formation consisted of two charge–discharge cycles at a rate of 0.2 C. Charging was conducted in constant-current/constant-voltage (CCCV) mode to an upper cut-off voltage of 4.2 V, with the voltage held constant until the current dropped below 0.05 C. Discharging followed in constant-current (CC) mode to a lower cut-off voltage of 3.0 V. For electrolyte analysis after extended cycling, cells were degassed and subjected to 200 cycles at 1 C under the same operating conditions used during formation. All cycling procedures were carried out on a Maccor 4000 battery tester in a temperature-controlled chamber maintained at 20 °C. After formation or long-term cycling, the cells were opened in a glovebox (O_2_ ≤ 0.1 ppm/H_2_O < 2.0 ppm) and the aged electrolyte was extracted via centrifugation of the negative electrode and separator. For a detailed description of the extraction process the reader is kindly referred to previous studies (Horsthemke et al. 2020).

### Micronucleus assay with fluorescence microscopic evaluation

In vitro genotoxicity in the human liver carcinoma cell line HepG2 was assessed using the MN assay. The assay was performed according to OECD TG 487 (OECD 2023) and as described by Schreiber et al. (2025). Cells were grown in Dulbecco’s Modified Eagle Medium (DMEM, high glucose), supplemented with 10% (*v*/*v*) of fetal bovine serum (FBS), 100 units/mL penicillin and 0.1 mg/mL streptomycin at 37 °C and 5% CO_2_ in a humidified atmosphere. 400 000 HepG2 cells in 5 mL DMEM with supplements were seeded on Polysine® slides (Thermo Fisher Scientific) in Quadriperm® dishes (Sarstedt, Nümbrecht, Germany). Test substances were dissolved in dimethyl sulfoxide (DMSO) and diluted with DMEM without FBS to a final concentration of 0.5% (*v*/*v*) DMSO and 250–5 000 ppm electrolyte, 50–500 µM pure additive or 500 µM ring-opening product. Mitomycin C (0.6 µM, Carl Roth) was used as a genotoxic positive control (PC), pure DMSO as a negative control (NC). At least 500 cells per slide were counted for their number of nuclei to determine the cytotoxicity of the test substance, and no less than 1 000 binucleate cells to evaluate the percentage of binucleate cells with micronuclei (MN). If the percentage of MN (Eqn. S1) was significantly different (according to Student’s two-sample *t*‑test) from the corresponding NC, a substance was considered to be genotoxic, while concentrations with cytotoxicity above 60% were neglected for the evaluation. Three biological replicates (*n* = 3) were performed each.

### Micronucleus assay using flow cytometry

A flow cytometric protocol was carried out for an alternative detection of MN (Avlasevich et al. 2006; Bryce et al. 2011; Nüsse und Kramer 1984). For this purpose, 15 000 HepG2 cells in 200 µL DMEM with supplements were seeded per well in sterile 96-well-plates (Sarstedt) and cultured for 24 h under cell culture conditions. For the determination of cytotoxicity, the cell count prior to substance incubation was required. For this purpose, after 24 h of cell growth, eight wells on an additional plate were stained and lysed identical to the substance incubation plate (described in the following). Contemporaneously, the test substances were dissolved in DMSO and diluted with DMEM with supplements to a final DMSO concentration of 0.5% (*v*/*v*) for substance incubation. The final concentrations in the assay were 250–5 000 ppm for the electrolytes and 80–500 µM for the additives and the ring-opening products. The medium was aspirated and 200 µL of the incubation solutions were transferred to each well. Pure DMSO (0.5%, *v*/*v*) and etoposide (1 µM, Cayman Chemical, Ann Arbor, US) were included as NC and PC, respectively. Incubation under cell culture conditions followed for 40 h. To stop cell proliferation, the plates were incubated on ice for 20 min before starting the staining procedure. The incubation solution was replaced by 50 µL ethidium monoazide bromide (EMA, 20.22 µM) in PBS/FBS (49/1, *v*/*v*), and the plates were placed on ice again. The plate cover was removed to enable the use of a visible light source for dye activation for 30 min. Following this step, work was conducted in the dark. 150 µL ice-cold PBS/FBS (49/1, *v*/*v*) mixture were added to each well, before the entire liquid was removed and replaced by 100 µL lysis solution I (NaCl 10 mM, sodium citrate dihydrate 3.4 mM, IGEPAL® 0.03%, RNase A 0.5 mg/mL, SYTOX® green 0.4 µM). After 1 h of incubation under standard conditions, 100 µL of lysis solution II (sucrose 250 mM, citric acid monohydrate 78 mM, SYTOX® green 0.4 µM) were added per well. The plates were shaken carefully to mix the solutions, and covered with an adhesive film. After 30 min of incubation in the dark at room temperature, the plates were measured at once or within four days after storage at 4 °C. To ensure a homogeneous distribution, cells were resuspended directly before measurement. A NovoCyte flow cytometer (Agilent, Waldbronn, Germany) with the corresponding software NovoExpress Version 1.3.0 was used for analysis of cell- and micronuclei. An Argon laser with an excitation wavelength of 488 nm was employed and emission detectors were used with wavelengths of 585/40 nm and 530/30 nm for EMA and SYTOX®, respectively. The flow rate of the sheath fluid NovoFlow® (Agilent, Waldbronn, Germany) was 35 µL/min, the plate was agitated for 10 s at 1500 rpm before sample acquisition, the system was rinsed every 3 wells and the stop conditions were either 5 000 cell nuclei or 75 µL sample. The events were classified as nuclei/micronuclei using a gating strategy based on six criteria (Avlasevich et al. 2006). A sample was regarded as genotoxic, if the micronucleus frequency (Eqn. S2) was ≥ 3 times the concurrent NC (Bryce et al. 2011, 2013). Test conditions that induced more than 60% cytotoxicity were not included in genotoxicity assessment. Three biological samples with four technical replicates each (*n* = 3 × 4) were carried out.

### Bacterial mutagenicity assay (Ames fluctuation test)

Mutagenicity was assessed using the Ames fluctuation test with *S. typhimurium* strains (TA97a, TA1537, TA98, TA100 and TA1535) without metabolic activation. The assay was based on the recommendations of ISO 11350 (ISO 2012) and OECD TG 471 (OECD 2020) and performed as described in Schreiber et al. (2025). The substances were dissolved in DMSO, and the final concentrations in the assay were 40–20 000 ppm for the electrolytes, 10–320 µM for the pure additives, and 10–1 000 µM for the ring-opening products. Pure DMSO (4% *v*/*v*, this DMSO concentration was also used for the samples) was included as NC, and individual mutagens for each strain as PCs (TA97a/TA1537: 10 µM Acridine Mutagen ICR 191 (ICR191), TA98: 3 µM 4‑Nitroquinoline N‑oxide (4-NQO) (both Sigma-Aldrich), TA100: 0.3 µM 4-NQO, TA1535: 280 µM N4‑Aminocytidin (N4-AC, Santa Cruz Biotechnology). For evaluation, the number of wells with revertants (yellow colour and/or visible bacterial colonies) was counted. A substance was regarded as mutagenic, if a dose dependency was observed and the mean number of revertants was at least twice the baseline of this specific experiment, see Eqn. S3.

### Software and statistics

Data analysis was performed using NovoExpress Version 1.3.0 (Agilent), Microsoft Excel 2021 (Redmond, WA, USA), and Origin 2024–25 (OriginLab Corporation, Northampton, MA, US). Statistical significance was evaluated by the two-sample Student’s *t*-test, and the data are presented as mean ± standard deviation (SD). Figures were created using ChemDraw 23.1.2.7 (Revvity Signals Software, Inc., Waltham, MA, US) and Inkscape 1.4 (The Inkscape Project, US).

## Results and discussion

The presented study investigated genotoxic and mutagenic effects of LIB electrolytes with sulphur-containing additives (PS, PES, DTD) in vitro. Considered were pure additives as well as electrolyte formulations at three electrochemical ageing stages and one suspected degradation product per additive. The main focus, however, was to study how toxicity changes with the electrochemical age of a LIB.

### Genotoxic effects on HepG2 cells

The genotoxic potential of the electrolytes, pure substances and decomposition products was investigated in HepG2 cells using the MN assay both with optical evaluation at the fluorescence microscope (FM) (OECD 2023) and with flow cytometry (FCM) (Avlasevich et al. 2006). In line with the OECD protocol, three concentrations were chosen for each substance and ageing state in the FM assay, while four concentrations of each substance were used in the less time-intensive FCM procedure. Despite the cytotoxicity data generated by the resazurin assay, it was necessary to evaluate the cytotoxicity in the experiment itself, which was done in the FM assay by calculating the replicative index (RI, applicable if cytochalasin B (CytB) was used in sample preparation, Eqn. S4) and in the FCM assay by calculating the relative increase in cell counts (RICC, applicable if CytB was not used, Eqn. S5) (OECD 2023). Concentrations with a RI/RICC below 40% were not considered for genotoxicity assessment due to the possibility of false positives.

### Fluorescence microscopic micronucleus assay

**Fig. 2:**
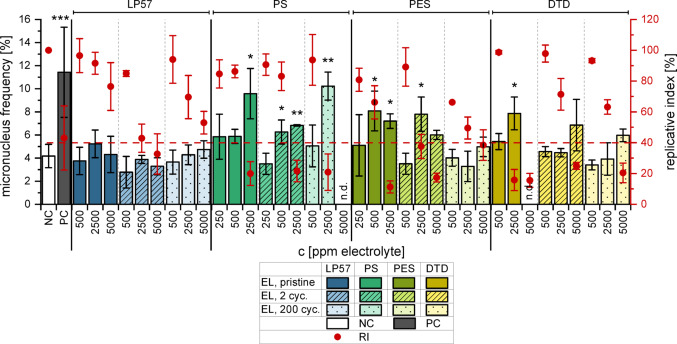
MN induction in HepG2 cells by EL_LP57_, EL_PS_, EL_PES_ and EL_DTD_ as detected with FM evaluation. The pristine electrolytes (blank) as well as samples after two (striped) and 200 (dotted) charge–discharge-cycles were analysed. In addition to the micronucleus frequency, the replicative index (RI) as a measure for cytotoxicity is illustrated. The dashed red line marks a RI of 40%. The mean ± SD of three independent experiments is shown for both values (n.d.: not detectable; PES 200 cycles, 5000 ppm: *n* = 2). DMSO (0.5%) and Mitomycin C (0.6 µM) were used as negative and positive controls, respectively. Significance levels of the MN frequencies were detemined using Student’s two-sample *t‑*test relative to the NC (* = *p* < 0.05, ** = *p* < 0.01, *** = *p* < 0.001)

No significant increase in MN was observed for EL_LP57_ (Fig. 2) and for the ring-opening products (Fig. S2). This indicates that the investigated additives were causing all further observed effects. Eight electrolyte samples supplemented with an additive showed a significant increase in MN frequencies. Due to the high cytotoxicity however, only two of those could decisively be labelled as genotoxic. First, EL_PS_ (2 cycles, 500 ppm, ~ 80 µM) showed simultaneously a sufficient RI (83%) and a significant increase in MN frequencies (6%). PS has been found to induce a dose-dependent increase of micronucleated reticulocytes in vivo in rats (Torous et al. 2000; Dertinger et al. 2011) and of MN in human lymphoblastoid cells in vitro (Bemis et al. 2016). Dertinger et al. (2011) and Kim et al. (2020) also demonstrated a PS-related increase in *Pig-a* mutation frequencies in vivo, an indicator of mutagenicity. The present study showed that PS was also genotoxic when part of LIB electrolyte, and that electrochemical ageing did not improve the toxic effects. However, only EL_PS_ after 2 cycles demonstrated clearly a genotoxic result. Even though pure PS, pristine EL_PS_ and EL_PS_ after 200 cycles showed a genotoxic increase in MN frequencies, it was accompanied by high cytotoxicity and thus unspecific effects could not be ruled out.

**Fig. 3:**
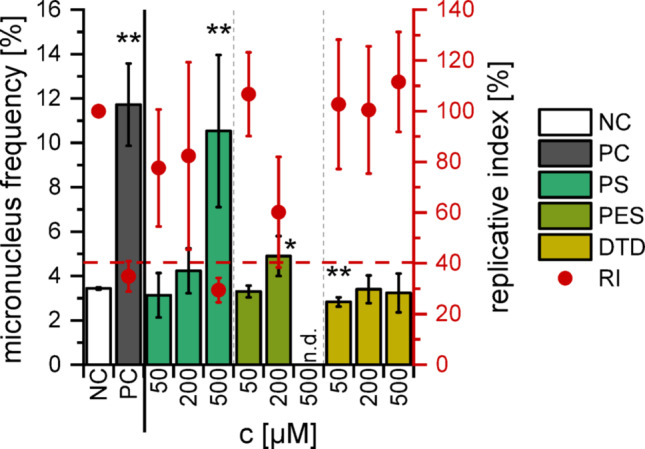
MN induction in HepG2 cells by the additives as detected with FM evaluation, as well as the corresponding replicative indices as a measure for cytotoxicity. The dashed red line marks a RI of 40%. The mean ± SD of three independent experiments is shown (n.d.: not detectable). DMSO (0.5%) and Mitomycin C (0.6 µM) were used as negative and positive controls, respectively. Significance levels of the MN frequencies were detemined using Student’s two-sample *t‑*test relative to the NC (* = *p* < 0.05, ** = *p* < 0.01)

The second genotoxic electrolyte sample according to this assay was the pristine EL_PES_ (500 ppm, ~ 80 µM) with a RI of 66% and 8% of binucleated cells with MN. To the best of our knowledge, there is a deep lack on PES genotoxicity data, but a comparison can be made to the pure substance also reported in this study. 200 µM PES induced significantly more MN than the solvent control while being not overly cytotoxic (Fig. 3).

PES has been proposed as an alternative to PS in LIB electrolytes and its use is not restricted e.g. in Europe (Tran et al. 2025; Regulation (EC) No 1272/2008). However, this falls short of the mark, as PES appears to act in the same way as PS from a toxicological point of view. Yet ageing of EL_PES_ is probably a means of detoxifying it in contrast to EL_PS_. This conclusion however cannot be drawn from the MN assay alone but needs further evaluation. The third additive DTD did not induce MN in the FM assay either with or without the electrolyte matrix (Fig. 2, Fig. 3). DTD has been shown to be carcinogenic after subcutaneous injection in mice (van Duuren et al. 1974), whereas mechanistic genotoxicity data is not available so far. However, this could indicate that higher concentrations of DTD would act genotoxic.

### Flow cytometric micronucleus assay

A flow cytometric MN assay (FCM) was performed because it offers various advantages like the possibility to study a broader concentration range, no subjectivity, the ability to perform technical replicates and the counting of a higher number of nuclei (both improving statistical reliability) as well as the additional generation of cell cycle data (Bryce et al. 2007). The results were compared to the ones of the FM assay.

**Fig. 4:**
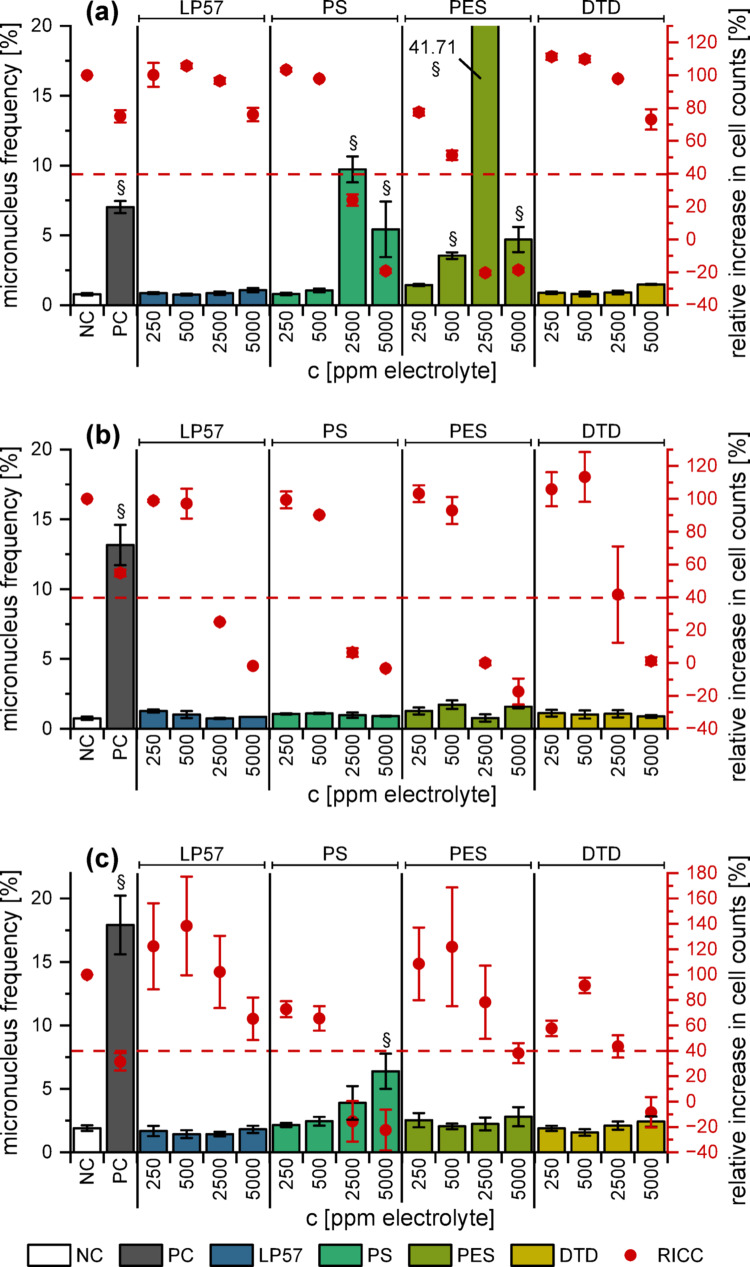
MN induction in HepG2 cells by the electrolytes as detected with FCM and their corresponding relative increases in cell counts (RICC). **(a)** pristine electrolyte, **(b)** electrolyte after 2 cycles and **(c)** after 200 cycles. The dashed red line marks the RICC level of 40%. The mean ± SD of three biological with four technical replicates each (*n* = 3 × 4) is shown. DMSO (0.5%) and Etoposide (1 µM) were used as negative and positive controls, respectively. Paragraph marks (§) indicate a MN frequency of ≥ 3 times the NC

In accordance with the FM assay, neither EL_LP57_ (Fig. 4) nor the ring-opening products (Fig. S3) showed substantial genotoxicity in the examined concentration range.

**Fig. 5:**
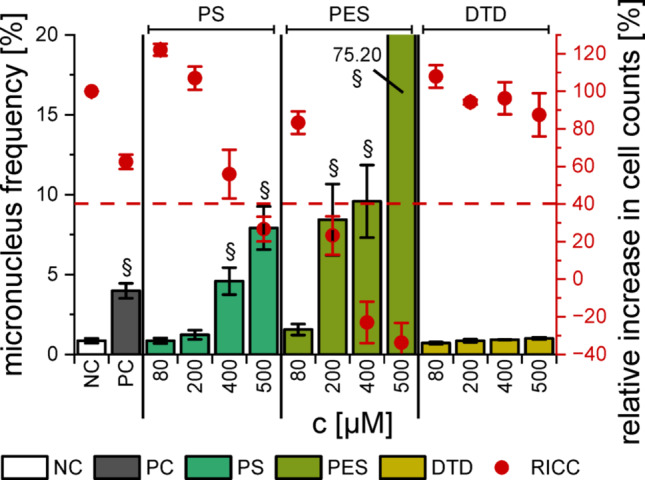
MN induction in HepG2 cells after the incubation with 80–500 µM of the pure additives as detected with FCM, and their corresponding relative increases in cell counts. The dashed red line marks the RICC level of 40%. The mean ± SD of three biological with four technical replicates each (*n* = 3 × 4) is shown. DMSO (0.5%) and Etoposide (1 µM) were used as negative and positive controls, respectively. Paragraph marks (§) indicate a MN frequency of ≥ 3 times the NC

The pristine and cycled EL_PS_ induced MN yet accompanied by a high cytotoxic potential. However, the concentration of 400 µM pure PS (~ 2500 ppm electrolyte) resulted in a distinct genotoxic effect (Fig. 5), which is in the same order of magnitude as the results of Kim et al. (2020), which were obtained in CHL (Chinese hamster lung) cells. The genotoxicity of PS has been well documented in literature, by means of both in vivo and in vitro studies (Kim et al. 2020; Hemminki 1983; Kaul 1985). In vitro it has been known to be accompanied by cytotoxicity, which increases with extended exposure (Kim et al. 2020). As the FCM assay involved longer incubation times than the FM method, this suggests that EL_PS_ is genotoxic in HepG2 cells but the genotoxic effect was disguised by cytotoxic events. The same could apply to the other substances.

For EL_PES_ however, the results of the FM and FCM assay were consistent. 500 ppm of the pristine electrolyte had a genotoxic effect in the FCM assay (Fig. 4a) as well as in the FM assay (Fig. 2), while the later ageing stages exhibited no genotoxicity. As discussed above, the enhanced detoxification during ageing is an argument in favour of PES.

Even so, electrolyte additives with even fewer toxic properties would be preferable. An example for such an additive could be DTD, as neither the pure substance (Fig. 5) nor EL_DTD_ at any ageing stage (Fig. 4) displayed genotoxic properties. This was consistent with the results of the FM assay but again includes the possibility of genotoxic events at higher concentrations, as DTD was shown to be carcinogenic in mice (van Duuren et al. 1974).

**Fig. 6:**
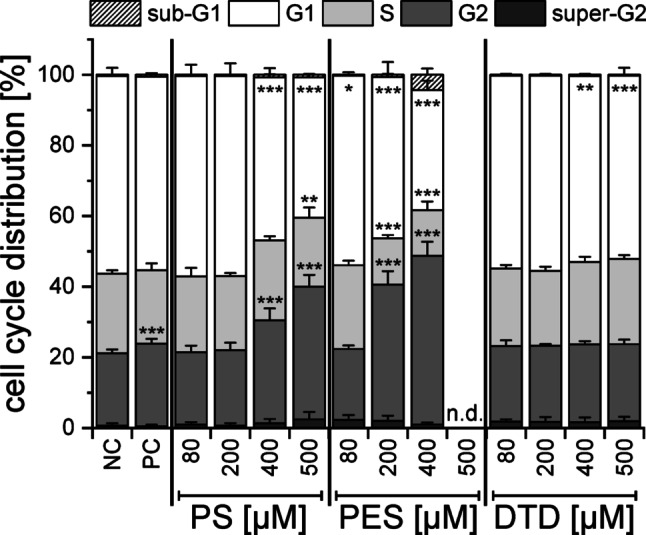
Cell cycle distribution of asynchronous HepG2 cells after 40 h of incubation with PS, PES or DTD (80–500 µM). DMSO (0.5%) and Etoposide (1 µM) were used as negative (NC) and positive controls (PC). The mean ± SD of three biological with four technical replicates each is shown (*n* = 3 × 4). Significance levels were detemined using Student’s two-sample *t*-test relative to the NC (* = *p* < 0.05, ** = *p* < 0.01, *** = p < 0.001) and are only presented for G1, S and G2 phase for reasons of clarity

Cell cycle data for the pure substances are presented in Fig. 6. DTD did not lead to prominent alterations in the cell cycle, whereas PS (≥ 400 µM) and PES (≥ 200 µM) showed decreased numbers of G1 cells (and S cells in case of PES) along with increased numbers of G2/M cells. Alkylating agents like the examined additives have been reported to induce an arrest at the G2/M checkpoint in both synchronized and asynchronous cell populations due to the inflicted DNA damage (Meyn und Murray 1984). For both PS and PES, the starting point for the increase of cells in G2/M phase was also the lowest concentration with increased MN. If the cell cycle arrest in the G2/M phase was observed at lower concentrations than the MN induction (Fig. S4 − S7), it would be always accompanied by cytotoxicity. Thus, a suppression of MN may be expected, but only in cases with high non-specific toxicity and not in subtoxic concentrations.

The cell cycle distributions of the electrolytes without additive resembled the negative control at all ageing stages. For EL_PS_, a significant increase in the number of G2/M cells was observed for all ageing stages at 2500 ppm, which was in line with the findings of the pure substance (2500 ppm ~ 400 µM). In general, the cell cycle distributions at 5000 ppm did not show a further increase in G2/M cells, but rather a decrease in this population. As these cell cycle results were accompanied by very high cytotoxicity, their validity is however questionable, also underlined by the fact that for some concentrations, the cell cycle results were not evaluable at all. Some concentrations of EL_PES_ showed altered cell cycle distributions for the pristine electrolyte and after formation, but not after 200 cycles. Pure PES as well as EL_PES_ (pristine and 2 cycles, but not 200 cycles) showed hints of apoptosis due to increased sub-G1-fractions. This served as another hint towards the detoxification of PES during cycling. EL_DTD_ imposed very little change on the cell cycle in all ageing stages. Also, no effect on the cell cycle could be observed for any of the assumed degradation products (Fig. S7).

### Mutagenicity in the bacterial reverse mutation assay

**Fig. 7:**
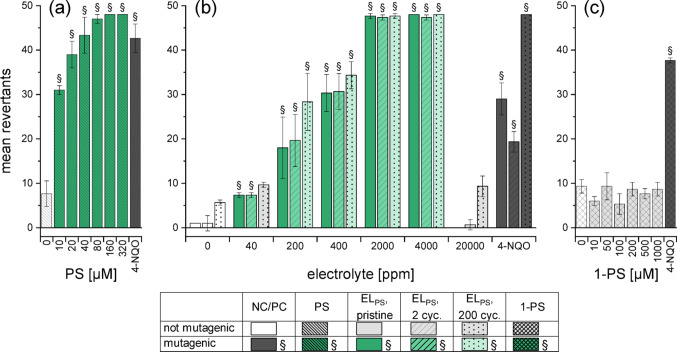
Mutagenicity of PS in *S. typhimurium* TA100, assessed with the Ames fluctuation test and expressed as mean revertants. The following substances were studied: **(a)** pure PS, **(b)** the three ageing stages of EL_PS_ and **(c)** pure 1‑PS. DMSO (4%) and 4‑Nitroquinoline N‑oxide (4-NQO, 0.3 µM) were used as negative and posititve controls, respectively. The presented data are the mean ± SD of the experiments conducted in triplicate. Paragraph marks (§) and coloured columns indicate an increase of ≥ 2 over the corresponding baseline

**Fig. 8:**
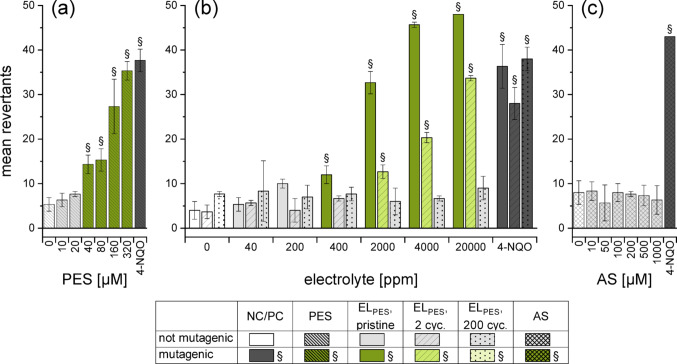
Mutagenicity of PES in *S. typhimurium* TA100, assessed with the Ames fluctuation test and expressed as mean revertants. The following substances were studied: **(a)** pure PES,** (b)** the three ageing stages of EL_PES_ and **(c)** pure AS. DMSO (4%) and 4‑NQO (0.3 µM) were used as negative and posititve controls, respectively. The presented data are the mean ± SD of the experiments conducted in triplicate. Paragraph marks (§) and coloured columns indicate an increase of ≥ 2 over the corresponding baseline

**Fig. 9:**
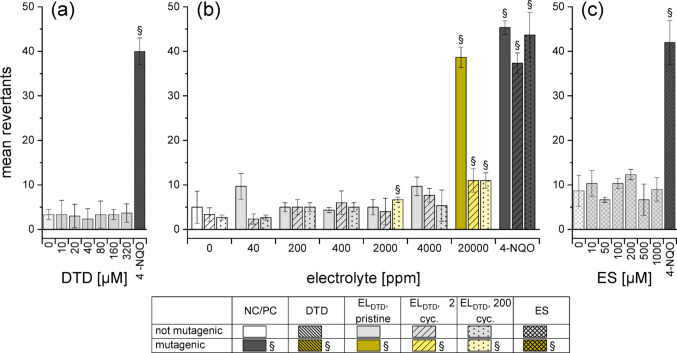
Mutagenicity of DTD in *S. typhimurium* TA100, assessed with the Ames fluctuation test and expressed as mean revertants. The following substances were studied: **(a)** pure DTD,** (b)** the three ageing stages of EL_DTD_ and **(c)** pure ES. DMSO (4%) and 4‑NQO (0.3 µM) were used as negative and posititve controls, respectively. The presented data are the mean ± SD of the experiments conducted in triplicate. Paragraph marks (§) and coloured columns indicate an increase of ≥ 2 over the corresponding baseline

Following the genotoxicity assessment, mutagenicity of the electrolytes was evaluated using the bacterial reverse mutation assay (Ames fluctuation test) in four different *S. typhimurium* strains each. Tests were conducted without metabolic activation, as PS is a known ultimate mutagen which was also used directly in the literature (IARC 2017). Table 3 presents an overview of the results of all strains and samples, showing whether a sample induced mutagenicity in a strain or not. Dose-depended results for the different pure substances and electrolytes are presented in detail for TA100 (Fig. 7 − 9), as this strain proved to be the most suitable for visualizing the differences between the additives. Results for the other strains are available in Fig. S8–S17.

**Table 3:** Mutagenicity in the Ames fluctuation test in four different *S. typhimurium* strains. The pure substances and (assumed) degradation products were analysed as well as the electrolyte without additives and the electrolytes with 2 wt% of the respective additives at three different ageing stages. Colour and symbol show evidently mutagenic samples (+), samples that were not mutagenic in the studied concentration range (-) and samples that were classified as not mutagenic although individual concentrations exceeded the baseline at low levels (o)

	Pure additives				Pristine Electrolyte				Electrolyte after 2 cycles				Electrolyte after 200 cycles				Degradation products			
	TA100	TA1535	TA98	TA1537^1^	TA100	TA1535	TA98	TA97a	TA100	TA1535	TA98	TA97a	TA100	TA1535	TA98	TA97a	TA100	TA1535	TA98	TA97a
LP57^2^	x	x	x	x	−	−	-	o	-	-	-	-	-	o	-	-	x	x	x	x
PS	+	+	-	-	+	+	-	-	+	+	-	-	+	+	-	+	-	-	-	-
PES	+	+	-	-	+	+	+	+	+	+	-	o	-	o	-	-	-	-	-	-
DTD	-	-	-	-	+	+	-	-	+	+	-	-	+	+	-	-	-	-	-	-

^1^ TA1537 replaced TA97a due to availability of bacteria

^2^ LP57 line: not applicable (x)

EL_LP57_ did not induce mutagenicity in any of the strains in either of the analysed conditions (Table 3, Fig. S8). Positive results observed in the following experiments could thus be attributed to the used additives or their degradation products.

### Mutagenicity of PS

PS caused base-pair substitutions (BPS) without metabolic activation (revertant bacteria in TA100, see Fig. 7, and TA1535, see Fig. S11), while no frameshift (FS) mutations in TA1537 and TA98 were induced (Fig. S9–S10). The type of mutations triggered were in accordance with the findings of Braun et al. (1977) and Simmon (1979). In both positive strains, a dose-dependency was observed, with TA1535 showing a slightly higher sensitivity to PS, which agrees with literature (Bartsch et al. 1983). For both BPS-detecting strains, the strength of the effect was comparable between pure substance and all electrolyte ageing stages (Fig. 7). The absence of TA100-revertants in the highest concentration of EL_PS_ was an indication of cytotoxicity, which was not observed for the pure additive due to a narrower concentration range.

1‑propanesulfonate (1‑PS) as an electrochemical degradation product of PS (Peschel et al. 2023) was not mutagenic under the conditions of the assay (Fig. 7, Fig. S9–S11). This and the absence of changes in mutagenicity during cycling leaded to the conclusion that PS is neither fully degraded nor incorporated into the passivation layers of the electrodes but was probably present in the electrolyte even after 200 cycles. Judging by the strength of the mutagenicity, in fact most PS seemed to remain stable in the electrolyte. Furthermore, after 200 cycles, low mutagenic effects were observed for EL_PS_ in TA97a in the highest concentrations, indicating that also FS mutations were possibly induced by PS, its degradation products or synergistic effects. Kim et al. (2020) demonstrated FS for very high concentrations of PS, however TA1537 (can be used instead of TA97a) being more sensitive towards PS than TA98. This was confirmed in the present study, where no mutations were observed in TA98.

### Mutagenicity of PES

The second sultone, PES, induced mutations in the same strains as PS (Table 3, Fig. 8, Fig. S12–S14) which leaded to the conclusion that BPS were provoked by PES in the investigated concentration ranges. The observed mutagenicity of PES was lower than of PS. Another remarkable difference between the two additives were the results of the cycled electrolytes as seen in Fig. 8: in contrast to EL_PS_, the mutagenicity of EL_PES_ decreased. While the results of the pristine EL_PES_ were comparable to the ones of the pure substance, the ability to induce BPS decreased as the number of cycles increased, resulting in little to no mutagenicity after 200 cycles. PES might have been more easily reduced electrochemically than PS due to the double bond (Tong et al. 2021; Li et al. 2012; Xia et al. 2014). Thus, a lower mutagenicity possibly resulted of less remnants of the initial additive in the aged electrolyte. PES could either have been incorporated into the SEI or degraded on the surface of the electrodes, leaving only degradation products in the electrolyte. A further difference between EL_PS_ and EL_PES_ lay in the results obtained for the strains detecting FS. Pristine EL_PES_ increased the number of revertants at 20 000 ppm in both TA97a and TA98. After formation and cycling, this effect decreased like the BPS induction. The assumed decomposition product allylsulfonate (AS) was not mutagenic in the evaluated strains and concentrations (Fig. S12–S14). To the best of our knowledge, there is no published mutagenicity data about PES, but mutagenic effects similar to PS are expected due to the related structures. However, the obtained data show that it evoked BPS to a lower extent than PS but produced more FS mutations.

### Mutagenicity of DTD

DTD showed a different picture than PS and PES. The pure substance was not mutagenic in any of the analysed strains up to 320 µM (Fig. 9, Fig. S15–S17). Braun et al. (1977) described no mutagenicity in FS detecting strains, however weak effects in TA1535 were found. These findings were confirmed in the following, as the concentrations used for assessing the electrolytes corresponded to a tenfold higher concentration than for the pure additive. In accordance with literature, only revertants in BPS detecting strains were induced by EL_DTD_ (Fig. 9, Fig. S15–S17). Weak mutagenicity was observed for some ageing stages even in concentrations that correspond to 320 µM DTD. A possible explanation for the slightly higher mutagenicity of EL_DTD_ in comparison with the pure substance is the instability of DTD in water (Kaiser et al. 1963). Either the electrolyte matrix could have had a stabilizing effect on DTD, or synergistic mutagenic effects with matrix components could have played a role. The number of revertants decreased dramatically after formation, however stagnated afterwards (Fig. 9), leaving EL_DTD_ still mutagenic after 200 cycles. Thus, the decrease in mutagenicity was less pronounced than for EL_PES_, yet the overall hazard of EL_DTD_ was still lower, as higher concentrations were needed for the same effect and (for TA100) because of the abrupt decrease in mutagenicity after formation. FS were not observed for any sample containing DTD, nor any mutagenic events for the assumed ring-opening product ethylsulfate (ES) (Fig. 9, Fig. S15–S17).

## Conclusion

Lithium-ion batteries play an important role in modern society and will increase in number as the transition away from fossil fuels progresses and the range of possible applications expands. The objective of this study was to investigate the genotoxic and mutagenic properties of LIB electrolytes with the sulphur-containing additives PS, PES and DTD. The genetic toxicity of the complete electrolyte formulations was addressed in this study for the first time, as was that of aged electrolytes.

The genotoxicity was assessed using two different versions of the in vitro micronucleus assay in HepG2 cells. The electrolyte without additives was not genotoxic at any stage of ageing, nor were the additive related degradation products, in both versions of the assay. In the fluorescence microscopic assay, one sample of electrolyte each with PS and PES were found to increase micronucleus frequency, while DTD was not genotoxic. In the flow cytometric assay, again samples containing PS and PES showed a genotoxic mechanism while DTD did not. Furthermore, the genotoxicity seemed to decrease during battery ageing. Cell cycle results obtained along with the fluorescence microscopic assay showed an arrest in the G2/M-phase for PS and PES, which occurred at the same concentrations as the increase in micronuclei.

The results of the bacterial reverse mutation test clearly indicated a mutagenic mode of action. The general trend in the mutagenicity of the studied substances was as follows: PS > PES > DTD. All electrolytes with additive induce base-pair substitutions, whereas frameshifts were only induced by PES. For PES and DTD, their mutagenic effects decreased with battery ageing, suggesting that detoxification by decomposition occurs. Although the mutagenic properties of EL_PS_ remained unchanged during ageing, the main degradation product was found to lack mutagenicity. This suggests that PS is either not fully degraded or is incorporated in the SEI or CEI in smaller quantities than the other additives.

In summary, the additive DTD is less genotoxic and mutagenic than PS and PES, and additionally is detoxified during battery ageing. For the use in LIBs, this makes DTD – from a toxicological point of view – clearly preferable to PS and PES. Moreover, for the first time it was proven in vitro that the toxicity of electrolytes is influenced by ageing due to degradation processes. State-of-the-art risk assessment of batteries relies on the classification of the raw materials, which is inadequate according to the results presented. Future investigations should therefore not fail to consider aged battery materials in the electrolyte and beyond.

## Supplementary Information

Below is the link to the electronic supplementary material.

Supplementary Material 1

## Data Availability

Additional data are presented in the Online Resource 1.
