# Supplementary material for: Effects of electrochemical ageing of lithium-ion battery electrolyte on its in vitro genotoxicity: a special focus on sultones
Source: Arch Toxicol. 2026 Jan 14;100(4):1589–602. doi: 10.1007/s00204-025-04246-2 (PMC13043621; doi:10.1007/s00204-025-04246-2)
Supplement: Supplementary file 1 — Supplementary Material 1 [file 204_2025_4246_MOESM1_ESM.pdf]

<u>**Supplementary Information**</u>

Effects of electrochemical ageing of lithium-ion battery electrolyte on its *in vitro* genotoxicity: a special focus on sultones

Elisabeth Christine Muschiol[a], Louisa Sophie Tölke[a], Christian-Timo Lechtenfeld[b], Thorsten Kuczius[c], Martin Winter[b,d], Sascha Nowak[b], Melanie Esselen[a]*

[a] Institute of Food Chemistry, University of Münster, Corrensstraße 45, 48149 Münster, Germany.

[b] MEET Battery Research Center, Institute of Physical Chemistry, University of Münster, Corrensstraße 46, 48149 Münster, Germany.

[c] Institute of Hygiene, University Hospital Münster, Robert Koch-Straße 41, 48149 Münster, Germany.

[d] Forschungszentrum Jülich GmbH, Helmholtz-Institute Münster, IMD-4, Corrensstraße 46, 48149 Münster, Germany.

Corresponding Author

*Prof. Dr. Melanie Esselen, Institute of Food Chemistry, 48149 Münster, Germany; e-mail address: esselen@uni-muenster.de

Introduction

One characteristic degradation product (Fig. ***S1***) for each additive was investigated in this study.

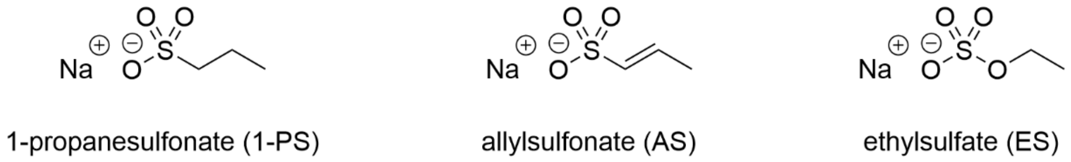

Fig. S1: Structures of the investigated electrochemical ring-opening products of the additives: 1-PS (for PS), ES (for DTD) and AS (for PES).

Experimental section

Eqn. S1: Calculation of the micronucleus frequency in the micronucleus assay with fluorescence microscopic evaluation.

$$MF = \frac{N_m}{N_b} \times 100$$

MF: micronucleus frequency [%]

N_m: number of binucleate cells with micronuclei

N_b: number of binucleate cells

Eqn. S2: Calculation of the micronucleus frequency in the flow cytometric micronucleus assay.

$$MF = \frac{N_m}{N_b} \times 100$$

MF: micronucleus frequency [%]

N_m: number of signals in the micronuclei-gate

N_b: number of signals in the cell nuclei-gate

Eqn. S3: Calculation of the baseline for each individual triplicate in the bacterial mutagenicity assay.

$$B = (M_p + SD_p)$$

B: baseline

M_p: mean revertants of the positive control

SD_p: standard deviation of the positive control

Results and discussion

Eqn. S4: Calculation of the replicative index in the micronucleus assay with fluorescence microscopic evaluation.

$$RI = \frac{(T_b + 2 \times T_m) \div T_t}{(C_b + 2 \times C_m) \div C_t} \times 100$$

RI: replicative index [%]

T_b: number of binucleate cells in the treated culture

T_m: number of multinucleate cells in the treated culture

T_t: total number of cells in the treated culture

C_b: number of binucleate cells in the respective untreated culture

C_m: number of multinucleate cells in the respective untreated culture

C_t: total number of cells in the respective untreated culture

Eqn. S5: Calculation of the relative increase in cell counts in the flow cytometric micronucleus assay.

$$RICC = \frac{T_e - T_0}{C_e - C_0} \times 100$$

$RICC$: relative increase in cell counts [%]

T_e: cell count at the end of substance incubation $(t = e)$ in the treated culture

T_0: cell count at the start of substance incubation $(t = 0)$ in the treated culture

C_e: cell count at the end of substance incubation $(t = e)$ in the respective untreated culture

C_0: cell count at the end of substance incubation $(t = 0)$ in the respective untreated culture

The genotoxicity of one characteristic degradation product per additive was assessed using both the fluorescence microscopic (Fig. **S*2***) and the flow cytometric (Fig. **S*3***) micronucleus assay.

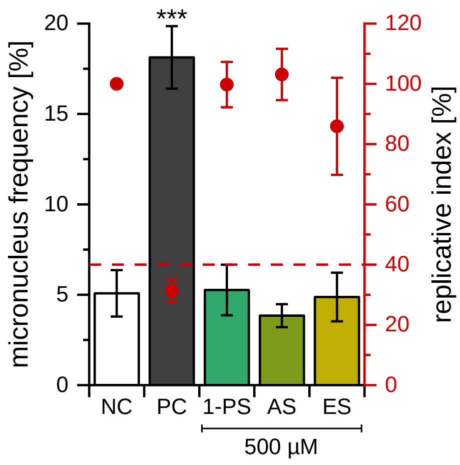

Fig. S2: MN induction in HepG2 cells after the incubation with 500 µM of the degradation products 1-propane sulfonate (1-PS), allyl sulfonate (AS) and ethyl sulfate (ES) as determined with fluorescence microscopic evaluation. The corresponding replicative index (RI) is depicted as well. The mean ± SD of three independent biological replicates is shown for both values. DMSO (0.5 %) and Mitomycin C (0.6 µM) were used as negative and positive controls, respectively. Significance levels of the MNi frequencies were detemined using Student's one-sample *t*-test relative to the NC (* = $p < 0.05$, ** = $p < 0.01$, *** = $p < 0.001$).

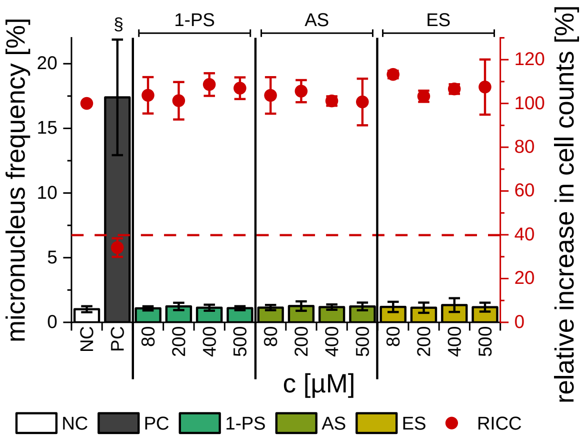

Fig. S3: MN induction in HepG2 cells after the incubation with 80–500 µM of the degradation products 1-PS, AS and ES as determined with flow cytometric evaluation. The corresponding relative increase in cell counts (RICC) is depicted as well. The mean ± SD of three independent biological replicates with four technical replicates each (n = 3 × 4) is shown. DMSO (0.5 %) and etoposide (1 µM) were used as negative and positive controls, respectively. Paragraph marks (§) indicate a MN frequency of ≥ 3 times the NC.

Contemporaneously with the flow cytometric assessment of micronuclei, cell cycle data was obtained (Fig. S4-7). Because the main aim of the assay was the determination of micronucleus frequencies, the positive control was selected based on its ability to trigger those rather than changing the cell cycle distribution.

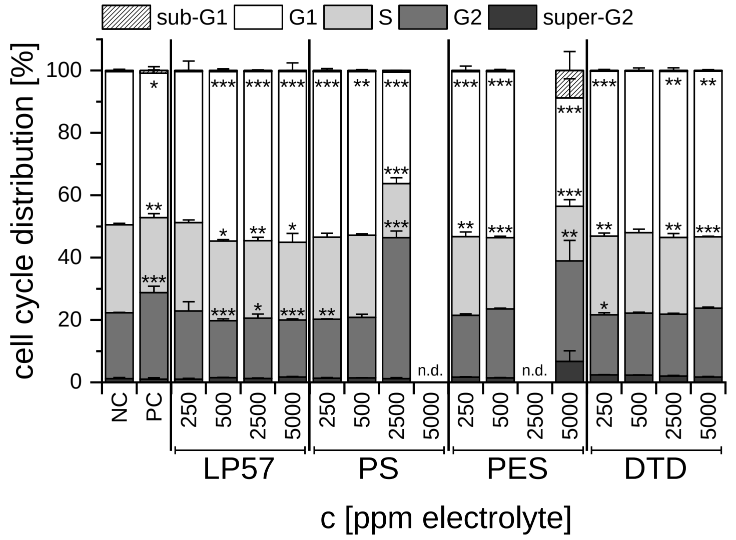

Fig. S4: Cell cycle distribution of HepG2 cells after 40 h of incubation with the pristine electrolytes (250–5000 ppm). DMSO (0.5 %) and Etoposide (1 µM) were used as negative (NC) and positive controls (PC). The mean ± SD of three biological with four technical replicates each is shown (n = 3 × 4, n.d.: not detectable). Significance levels were detemined using Student's one-sample *t*-test relative to the NC (* = $p < 0.05$, ** = $p < 0.01$, *** = p < 0.001) and are only presented for G1, S and G2 phase for reasons of clarity.

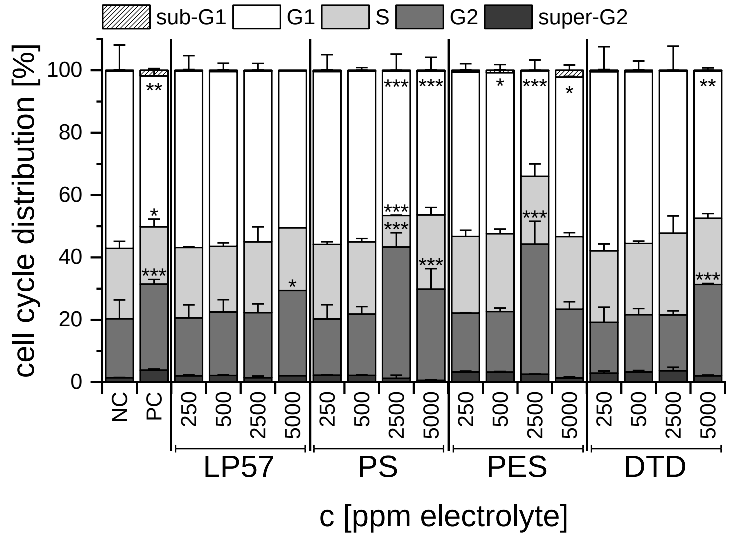

Fig. S5: Cell cycle distribution of HepG2 cells after 40 h of incubation with the electrolytes after formation (250–5000 ppm). DMSO (0.5 %) and Etoposide (1 µM) were used as negative (NC) and positive controls (PC). The mean ± SD of three biological with four technical replicates each is shown (n = 3 × 4). Significance levels were detemined using Student's one-sample *t*-test relative to the NC (* = $p < 0.05$, ** = $p < 0.01$, *** = p < 0.001) and are only presented for G1, S and G2 phase for reasons of clarity.

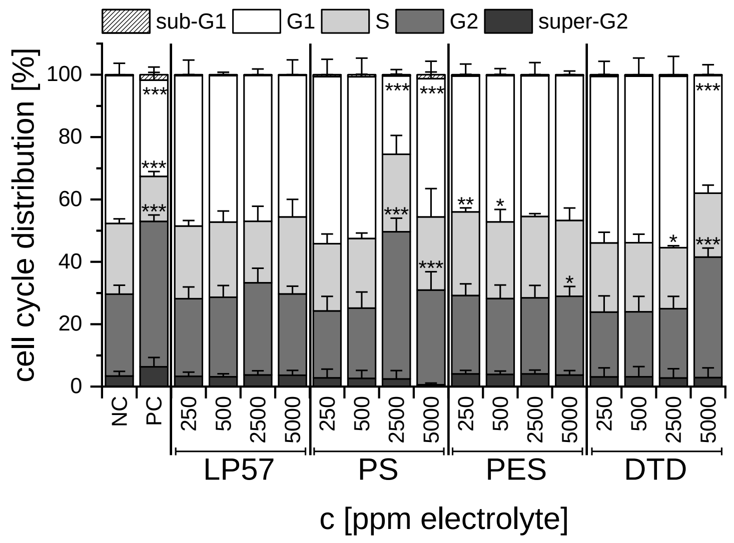

Fig. S6: Cell cycle distribution of HepG2 cells after 40 h of incubation with the electrolytes after cycling (250–5000 ppm). DMSO (0.5 %) and Etoposide (1 µM) were used as negative (NC) and positive controls (PC). The mean ± SD of three biological with four technical replicates each is shown (n = 3 × 4). Significance levels were detemined using Student's one-sample *t*-test relative to the NC (* = p < 0.05, ** = p < 0.01, *** = p < 0.001) and are only presented for G1, S and G2 phase for reasons of clarity.

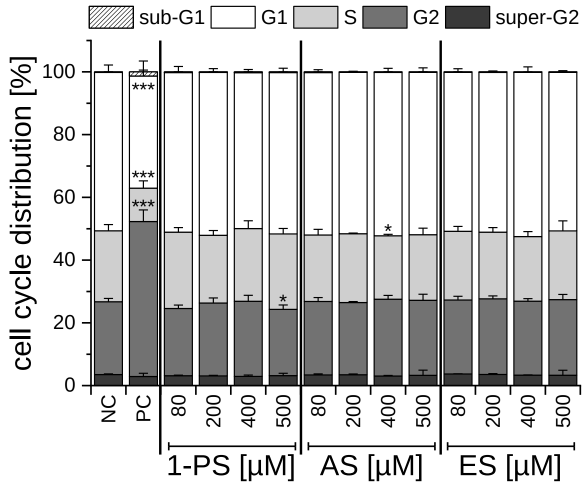

Fig. S7: Cell cycle distribution of HepG2 cells after 40 h of incubation with 1-PS, AS or ES (20–500 µM). DMSO (0.5 %) and Etoposide (1 µM) were used as negative (NC) and positive controls (PC). The mean ± SD of three biological with four technical replicates each is shown (n = 3 × 4). Significance levels were detemined using Student's one-sample *t*-test relative to the NC (* = *p* < 0.05, ** = *p* < 0.01, *** = p < 0.001) and are only presented for G1, S and G2 phase for reasons of clarity.

Mutagenicity was assessed in four different *S. typhimurium* strains for each set of samples. While the results of stain TA100 (electrolytes except EL_{LP57} and pure substances) are depicted in the main body of the paper, the results for TA97a/TA1537, TA98 and TA1535 are shown here in the SI. The mutagenicity of EL_{LP57} in all strains is shown in Fig. S8. All data for samples related to PS are depicted in Fig. S9–S11, data related to PES in Fig. S12–S14 and data related to DTD in Fig. S15–17.

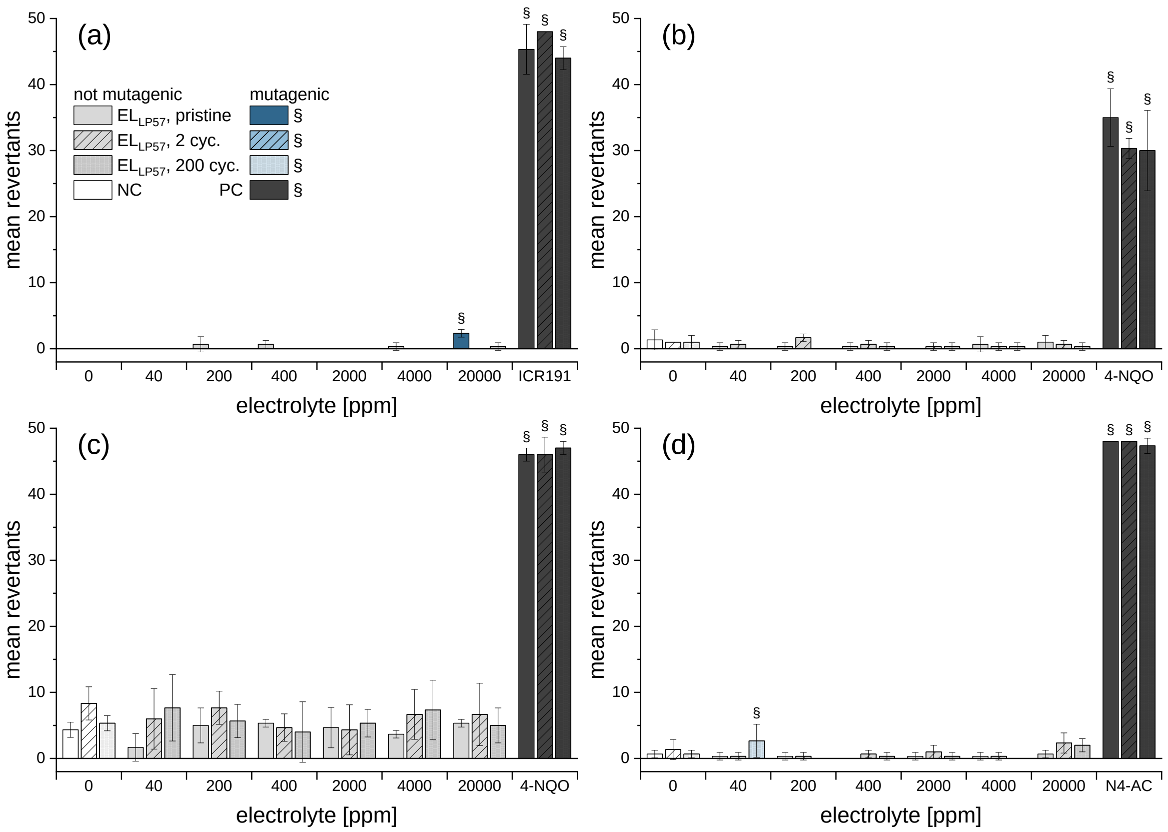

Fig. S8: Mutagenicity of EL_{LP57} in *S. typhimurium* **(a)** TA97a, **(b)** TA98, **(c)** TA100 and **(d)** TA1535, assessed with the Ames fluctuation test. The following substances were tested: pristine EL_{LP57} (blank), EL_{LP57} after 2 (striped) and 200 cycles (dotted). DMSO (4 %) and ICR 191, 4-NQO and N4-AC (for concentrations see section 2.5) were used as negative and posititve controls, respectively. The presented data are the mean revertants ± SD of the experiments conducted in triplicate. Paragraph marks (§) and coloured columns indicate an increase of ≥ 2 over the corresponding baseline. Even though revertants are detected in TA97a for the pristine electrolyte at 20 000 ppm, it is not considered to be mutagenic due to the fact that no mutagenicity was observed in the electrolyte with PS and DTD at the same concentration/ageing state/bacterial strain. In TA1535, EL_{LP57} after 200 cycles shows this increase at 40 ppm electrolyte but is not regarded as mutagenic due to the lack of dose-dependency.

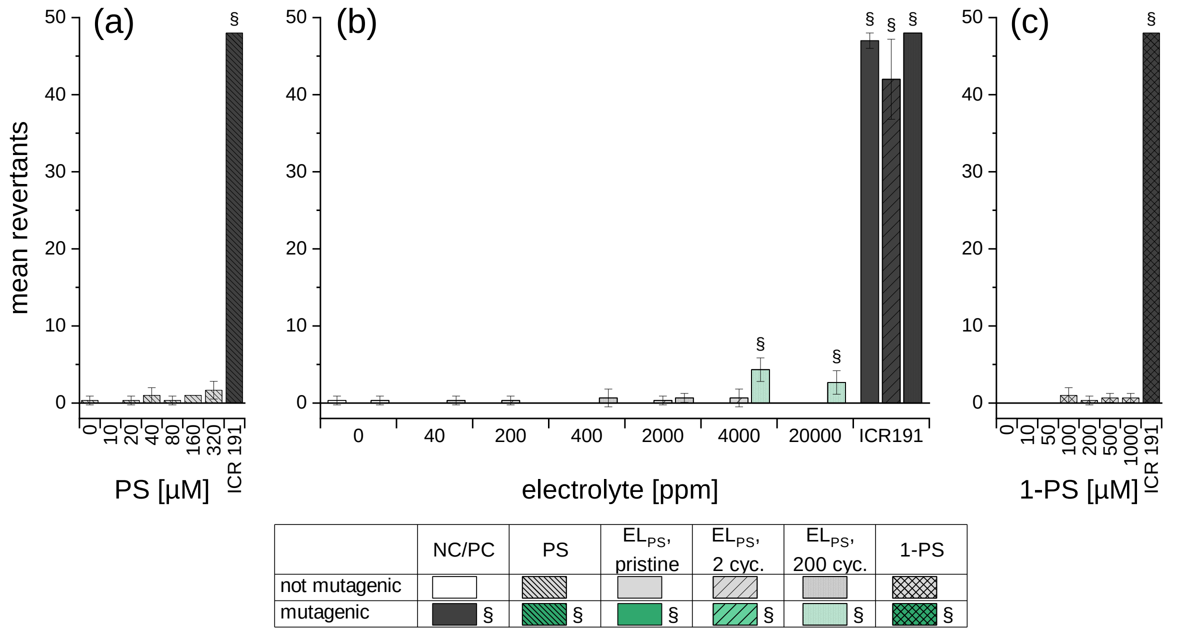

Fig. S9: Mutagenicity of PS in *S. typhimurium* TA97a (b and c) and TA1537 (a), assessed with the Ames fluctuation test. The following substances were tested: **(a)** pure PS, the three ageing stages of EL_{PS} and **(c)** pure 1-PS. DMSO (4 %) and Acridine Mutagen ICR 191 (ICR191, 10 µM) were used as negative and posititve controls, respectively. The presented data are the mean revertants ± SD of the experiments conducted in triplicate. Paragraph marks (§) and coloured columns indicate an increase of ≥ 2 over the corresponding baseline.

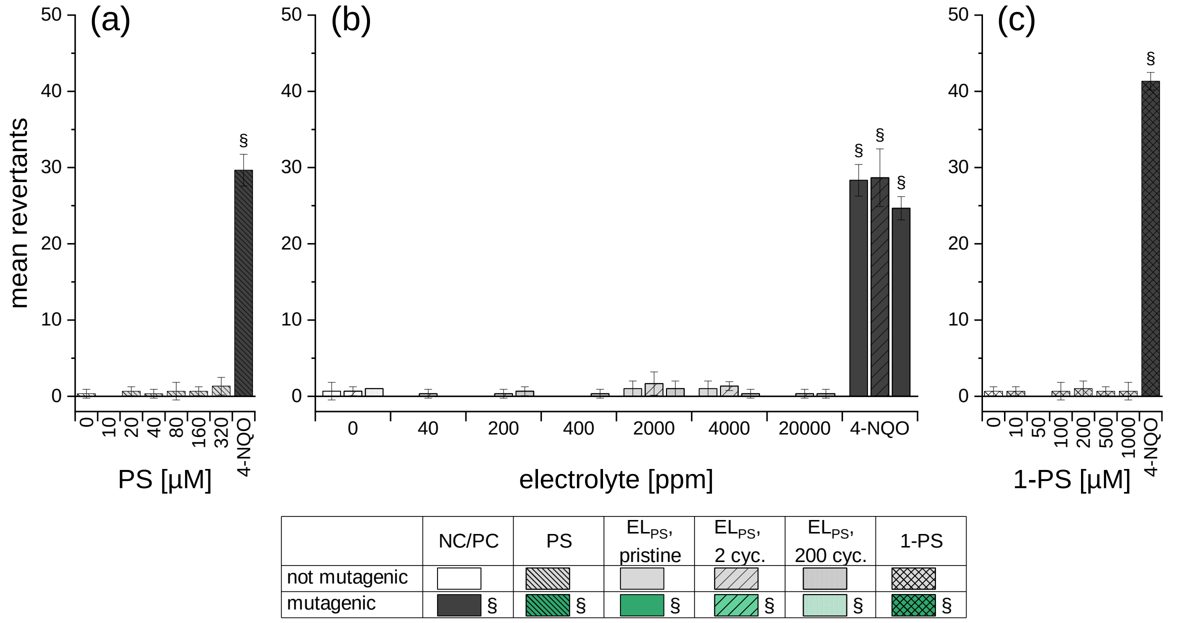

Fig. S10: Mutagenicity of PS in *S. typhimurium* TA98, assessed with the Ames fluctuation test. The following substances were tested: **(a)** pure PS, the three ageing stages of EL_{PS} and **(c)** pure 1-PS. DMSO (4 %) and 4-Nitroquinoline N-oxide (4-NQO, 3 µM) were used as negative and posititve controls, respectively. The presented data are the mean revertants ± SD of the experiments conducted in triplicate. Paragraph marks (§) and coloured columns ndicate an increase of ≥ 2 over the corresponding baseline.

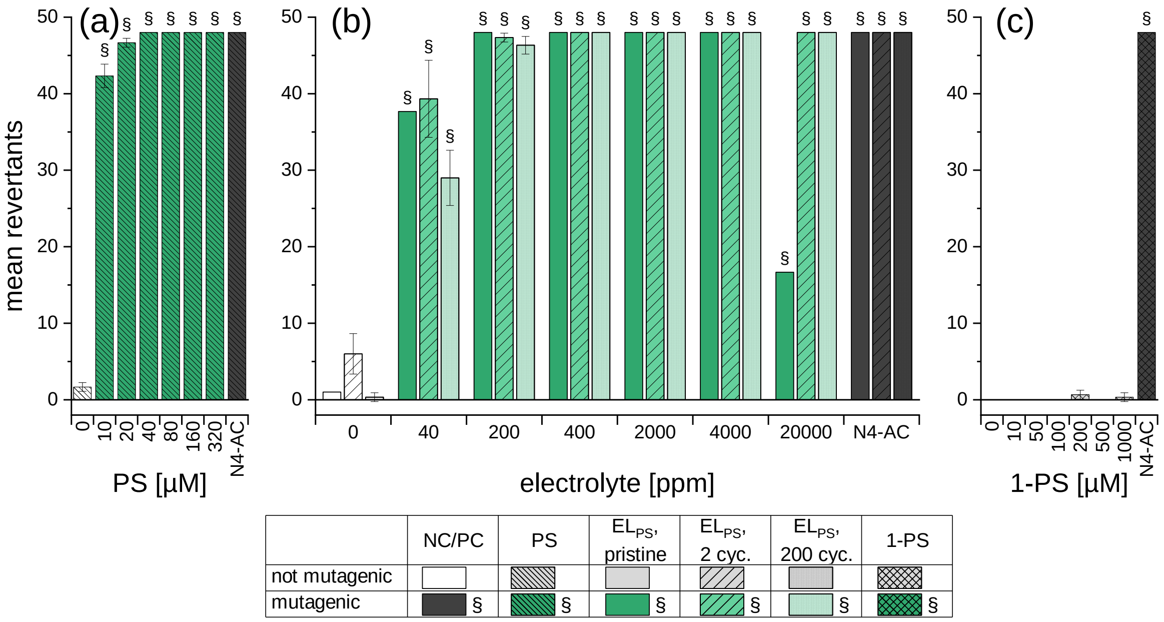

Fig. S11: Mutagenicity of PS in *S. typhimurium* TA1535, assessed with the Ames fluctuation test. The following substances were tested: **(a)** pure PS, the three ageing stages of EL_{PS} and **(c)** pure 1-PS. DMSO (4 %) and N4-Aminocytidin (N4-AC, 280 µM) were used as negative and posititve controls, respectively. The presented data are the mean revertants ± SD of the experiments conducted in triplicate. Paragraph marks (§) and coloured columns indicate an increase of ≥ 2 over the corresponding baseline.

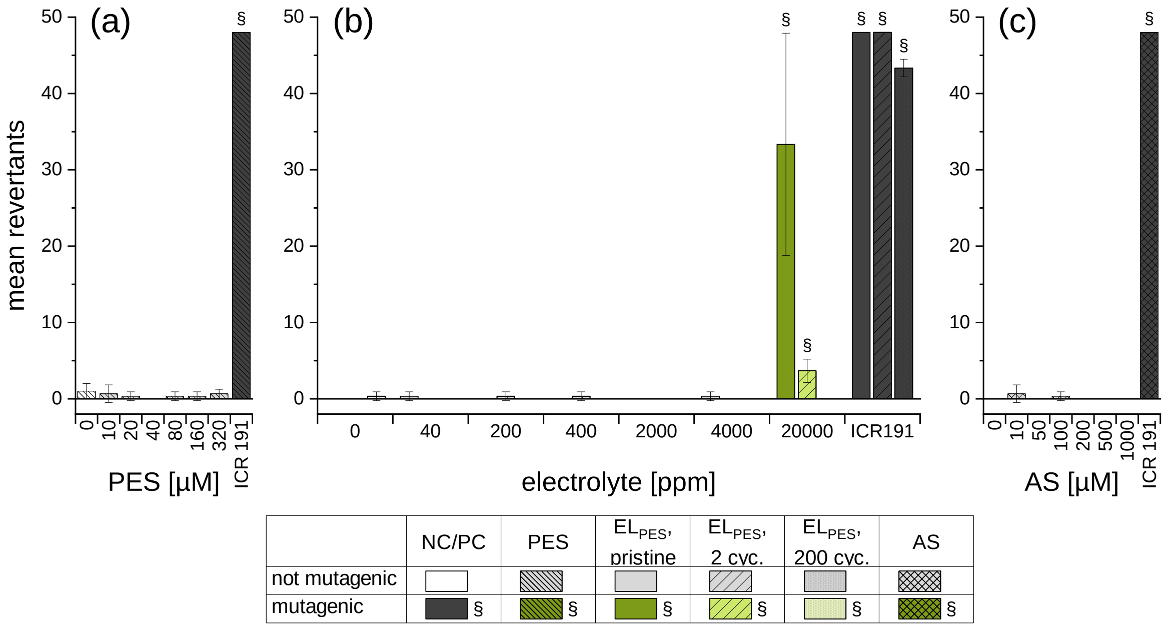

Fig. S12: Mutagenicity of PES in *S. typhimurium* TA97a (b and c) and TA1537 (a), assessed with the Ames fluctuation test. The following substances were tested: **(a)** pure PES, the three ageing stages of EL_{PES} and **(c)** pure AS. DMSO (4 %) and Acridine Mutagen ICR 191 (ICR191, 10 µM) were used as negative and posititve controls, respectively. The presented data are the mean revertants ± SD of the experiments conducted in triplicate. Paragraph marks (§) and coloured columns indicate an increase of ≥ 2 over the corresponding baseline.

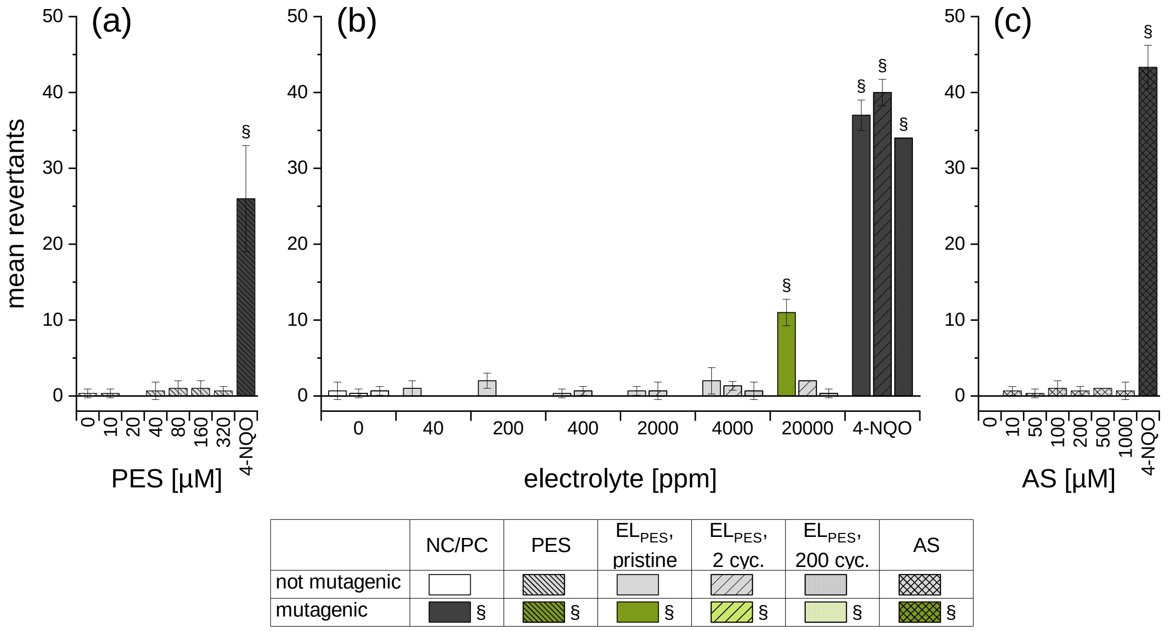

Fig. S13: Mutagenicity of PES in *S. typhimurium* TA98, assessed with the Ames fluctuation test. The following substances were tested: **(a)** pure PES, the three ageing stages of EL_{PES} and **(c)** pure AS. DMSO (4 %) and 4-Nitroquinoline N-oxide (4-NQO, 3 µM) were used as negative and posititve controls, respectively. The presented data are the mean revertants ± SD of the experiments conducted in triplicate. Paragraph marks (§) and coloured columns indicate an increase of ≥ 2 over the corresponding baseline.

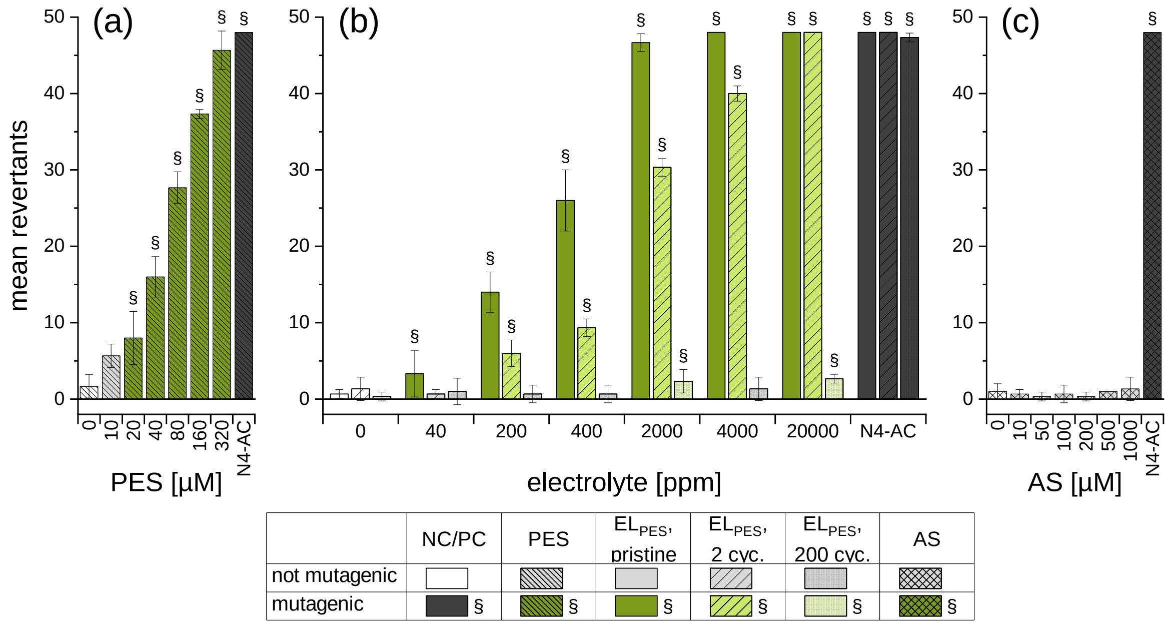

Fig. S14: Mutagenicity pf PES in *S. typhimurium* TA1535, assessed with the Ames fluctuation test. The following substances were tested: **(a)** pure PES, the three ageing stages of EL$_{PES}$ and **(c)** pure AS. DMSO (4 %) and N4-Aminocytidin (N4-AC, 280 µM) were used as negative and posititve controls, respectively. The presented data are the mean revertants ± SD of the experiments conducted in triplicate. Paragraph marks (§) and coloured columns indicate an increase of ≥ 2 over the corresponding baseline.

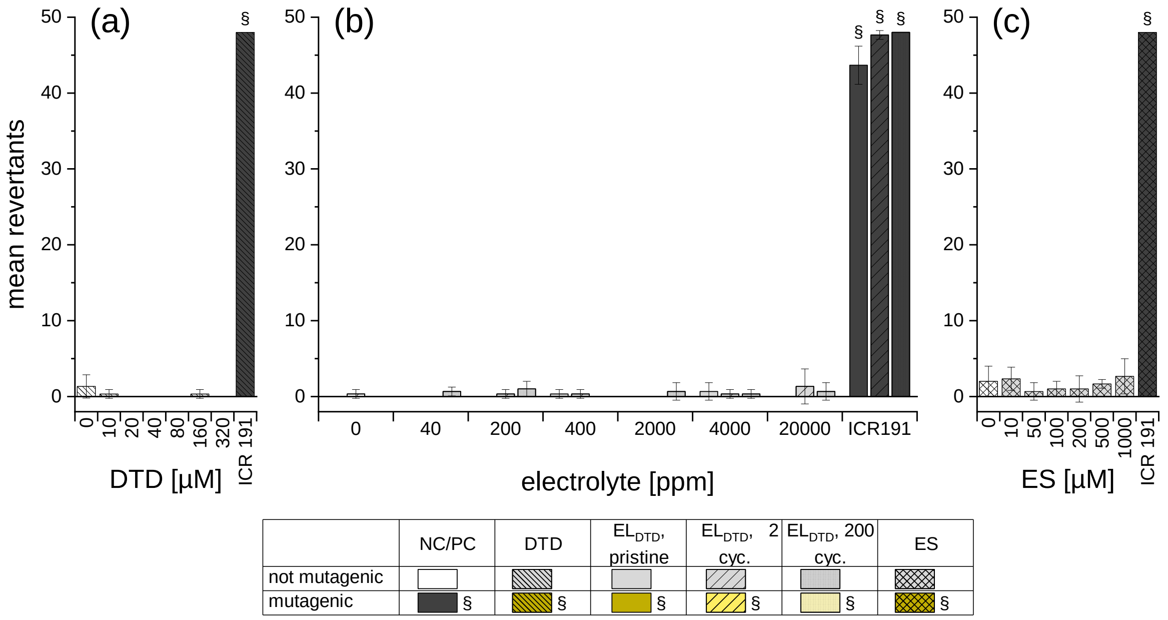

Fig. S15: Mutagenicity of DTD in *S. typhimurium* TA97a (b and c) and TA1537 (a), assessed with the Ames fluctuation test. The following substances were tested: **(a)** pure DTD, the three ageing stages of EL_{DTD} and **(c)** pure ES. DMSO (4 %) and Acridine Mutagen ICR 191 (ICR191, 10 µM) were used as negative and posititve controls, respectively. The presented data are the mean revertants ± SD of the experiments conducted in triplicate. Paragraph marks (§) and coloured columns indicate an increase of ≥ 2 over the corresponding baseline.

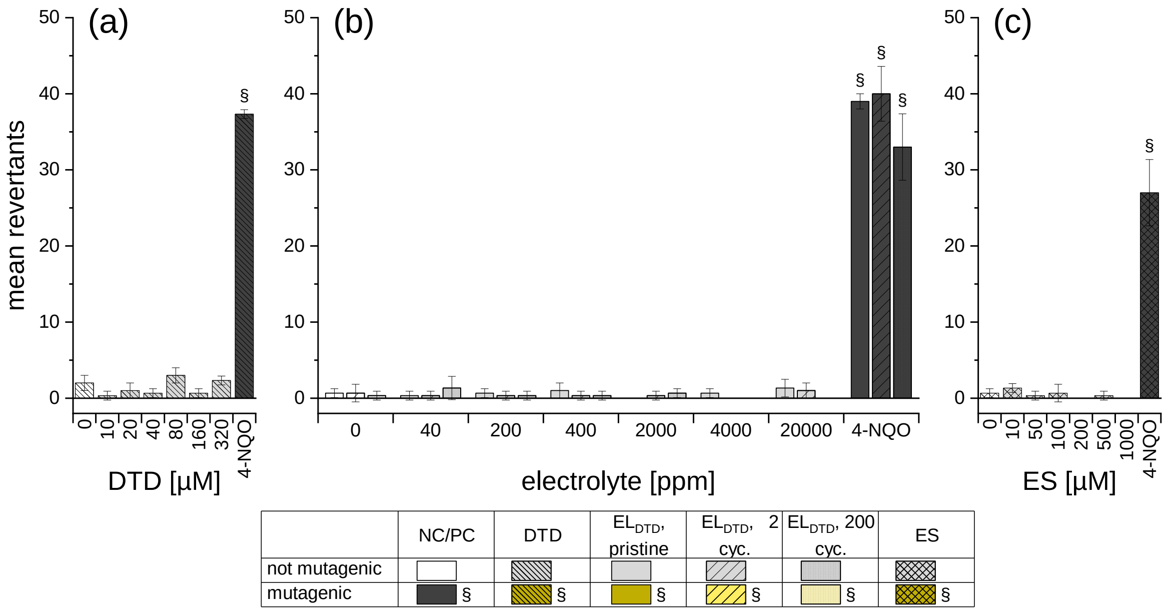

Fig. S16: Mutagenicity of DTD in *S. typhimurium* TA98, assessed with the Ames fluctuation test. The following substances were tested: **(a)** pure DTD, the three ageing stages of EL_{DTD} and **(c)** pure ES. DMSO (4 %) and 4-Nitroquinoline N-oxide (4-NQO, 3 µM) were used as negative and posititve controls, respectively. The presented data are the mean revertants ± SD of the experiments conducted in triplicate. Paragraph marks (§) and coloured columns indicate an increase of ≥ 2 over the corresponding baseline.

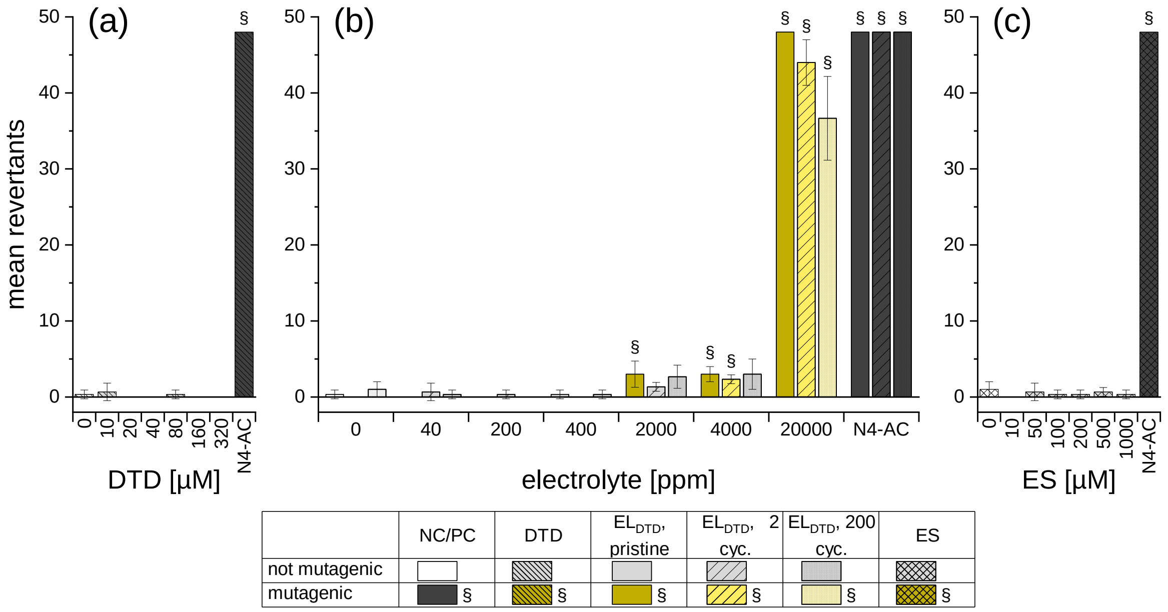

Fig. S17: Mutagenicity of DTD in *S. typhimurium* TA1535, assessed with the Ames fluctuation test. The following substances were tested: **(a)** pure DTD, the three ageing stages of EL_{DTD} and **(c)** pure ES. DMSO (4 %) and N4-Aminocytidin (N4-AC, 280 µM) were used as negative and posititve controls, respectively. The presented data are the mean revertants ± SD of the experiments conducted in triplicate. Paragraph marks (§) and coloured columns indicate an increase of ≥ 2 over the corresponding baseline.
